# Supplementary material for: Skeletal muscle methylome and transcriptome integration reveals profound sex differences related to muscle function and substrate metabolism
Source: Clin Epigenetics. 2021 Nov 3;13:202. doi: 10.1186/s13148-021-01188-1 (PMC8567658; doi:10.1186/s13148-021-01188-1)
Supplement: Supplementary file 1 — Additional file 1. Supplementary figures. S1-Correlation plots of chi2 tests of genomic locations. S2-Fibre type proportion analysis. S3-Quantile-quantile plot of −log10 transformed p-values for hormone meta-analysis in the Gene SMART study and sex meta-analysis in the three datasets included in the epigenome-wide association study. S4-Principal component analysis (PCA) of beta values of all tested CpGs across the participants in the FUSION, Gene SMART, and GSE38291 cohorts. S5-Distribution of the 10,000 random permutations for a negative correlation between DNA methylation and gene expression. S6-Gene expression for FOXO3 and ALDH1A1 validated in 3 cohorts. S7-Comparison of results from the full meta-analysis and from a meta-analysis excluding T2D participants in FUSION. [file 13148_2021_1188_MOESM1_ESM.docx]

**Supplementary Figure 1. Correlation plots of chi2 tests of genomic locations.** (**A**) Correlation plot of the percent contributions to the chi2 test for chromatin states in hyper-, hypo-, and non-DMPs. This plot is using the male chromatin state annotation in skeletal muscle but the female chromatin state annotation revealed equivalent results. Darker blue indicates a greater contribution to the significant relationship between DMP status and chromatin state. (**B**) Correlation plot of the percent contributions to the chi2 test for CGI status of hyper-, hypo-, and non-DMPs. Darker blue indicates a greater contribution to the significant relationship between DMP status and CGI status.


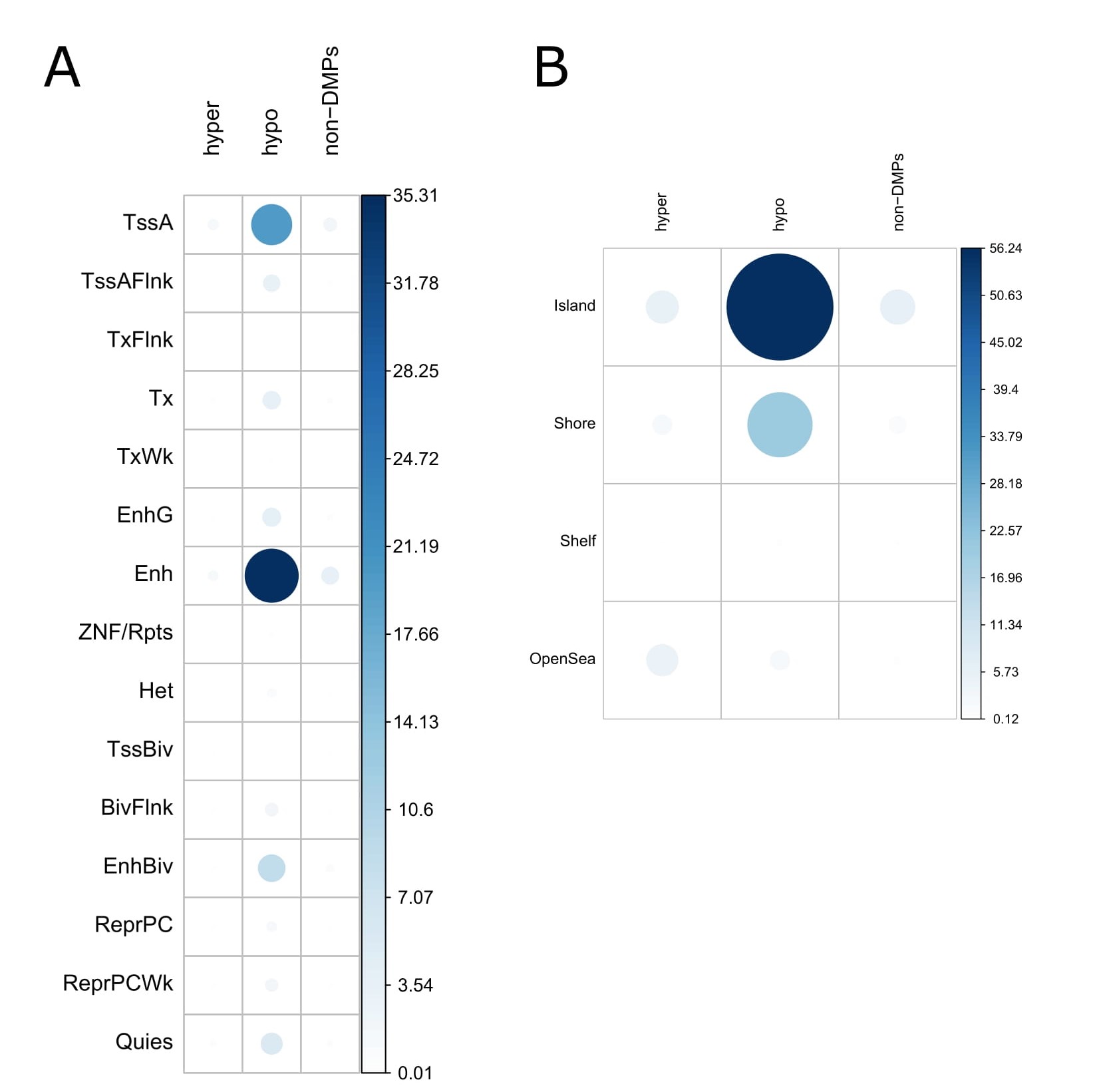

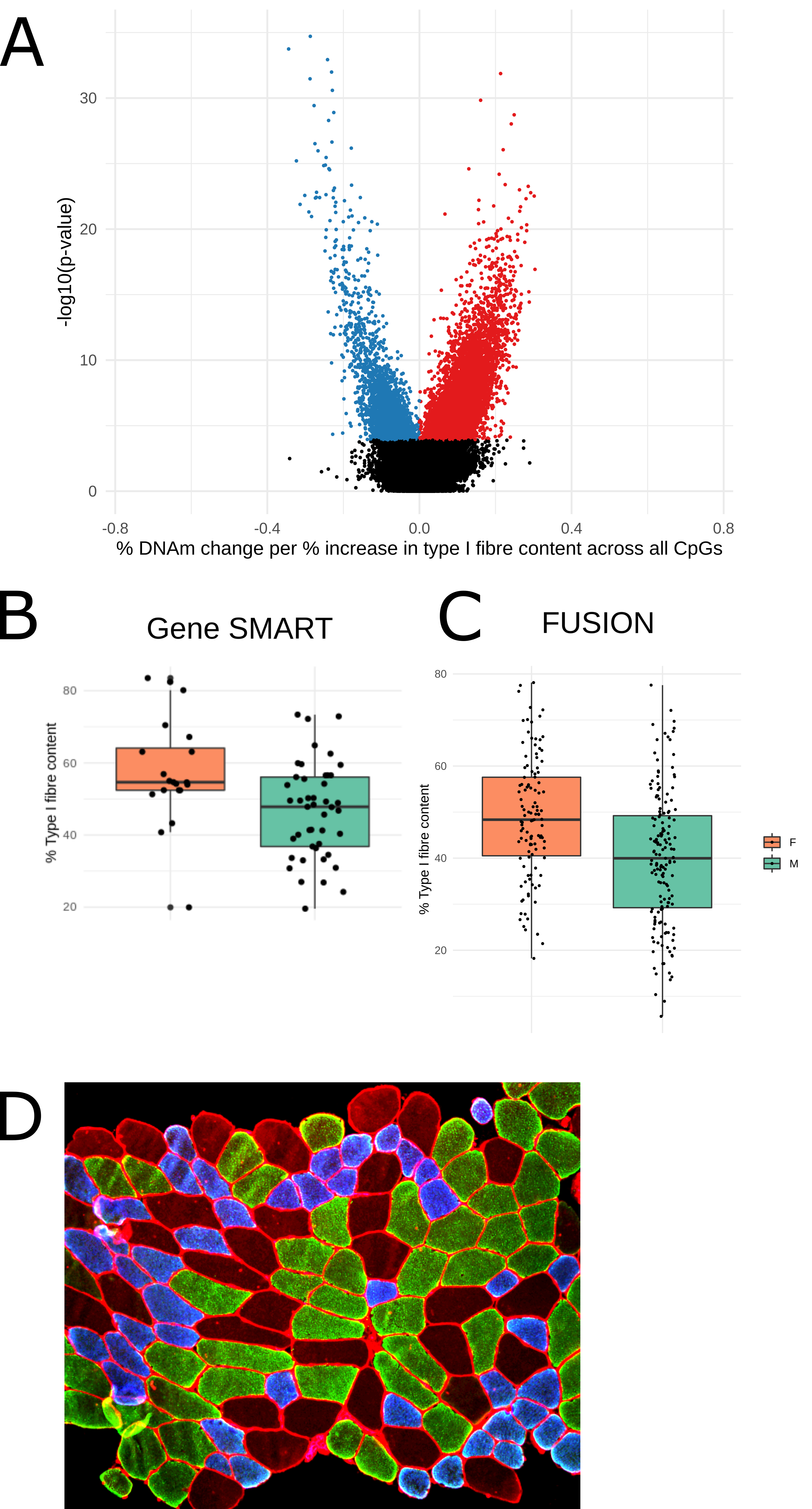


**Supplementary Figure 2. Fibre type proportion analysis. (A)** Differentially methylated positions (DMPs) with type I fibre proportion across all CpGs conducted with a meta-analysis of males and females, separately, from the Gene SMART and FUSION cohorts. Volcano plot of DNA methylation changes per percent increase in type I fibre content (expressed at percentage of beta value). Each point represents a tested CpG (665,904 in total) and those that appear in color are DMPs at a false discovery rate < 0.005; red DMPs are hypermethylated in type I fibres; blue DMPs are hypomethylated in type I fibres. The x-axis represents the amount of DNA methylation difference with increasing type I fibre content and the y-axis represents statistical significance (higher = more significant). **(B)** Percent of type I fibres in Gene SMART female and male muscle samples as determined by immunohistochemistry; p-value = 0.0001; 59% in females versus 47% in males. **(C)** Percent of type I fibres in FUSION female and male muscle samples as determined by RNA-seq; p-value = 2.06 x 10-7; 48.6% in females versus 39.9% in males. **(D)** Immunohistochemistry (IHC) myosin heavy chain staining of skeletal muscle section. Example of cross-sectional fibres of one participant. Blue fibres indicate type I, green indicate type IIa, and red indicate type IIx; cell membrane staining in red. A minimum of 100 fibres counted per person for approximation of fibre type proportions.

**Supplementary Figure 3. Quantile-quantile plot of −log_10_ transformed p-values for hormone meta-analysis in the Gene SMART study and sex meta-analysis in the three datasets included in the epigenome-wide association study.** Right panel using uncorrected p-values and left panel using bacon [1] bias- and inflation-corrected p-values. The genomic inflation factor is directly correlated with the expected number of true associations with the variable of interest. (**A**) QQ plots of all CpGs in DNA methylation analysis in females for the four hormones (estrogen, testosterone, free testosterone, and SHBG) and the first two principal components of the ovarian hormones (from follicle stimulating hormone, leutinizing hormone, estrogen, and progesterone). (**B**) QQ plots of all CpGs in DNA methylation analysis in males for the four hormones (estrogen, testosterone, free testosterone, and SHBG). QQ plots in **A** and **B** are did not show a separation of the observed from the expected, as all points are on or near the middle line between the x-axis and the y-axis; meaning that not many p-values are more significant than expected under the null hypothesis. (**C**) Contributors to the dimensions of the principal component analysis (PCA) of the ovarian hormones in females used in the linear model in **A** (PC1 and PC2). Larger circle indicates a larger contribution to the given dimension of the PCA. (**D**) QQ plots of all CpGs in DNA methylation of each study in the sex meta-analysis. QQ plots show a clear separation of the observed from the expected, as many points separate from the black line between the x-axis and the y-axis; meaning that many p-values are more significant than expected under the null hypothesis.





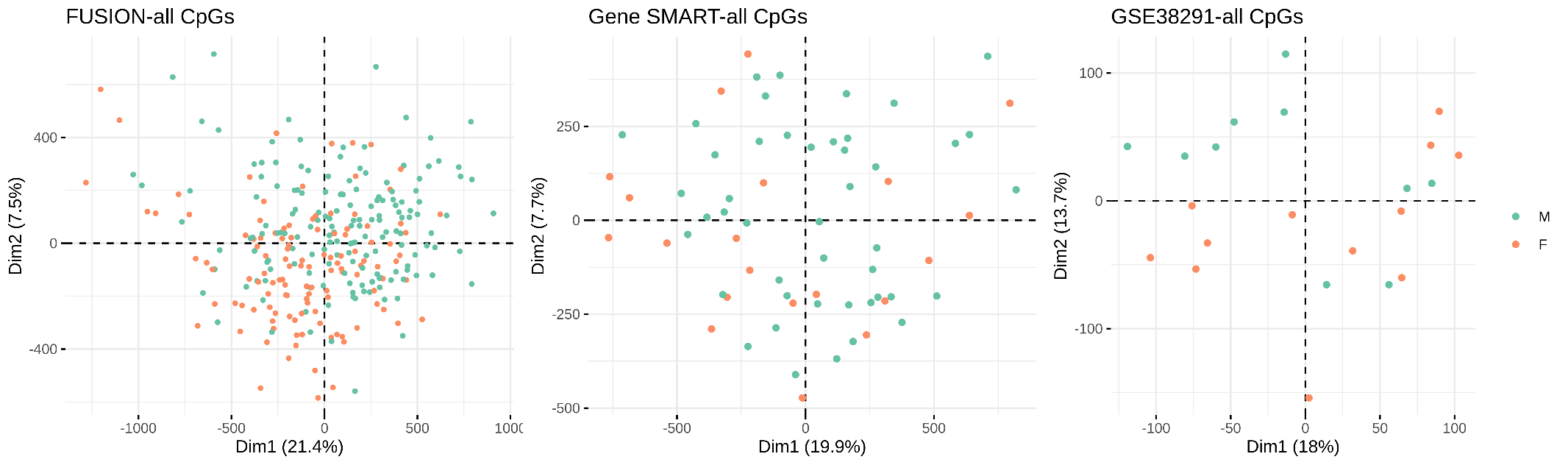


**Supplementary Figure 4. Principal component analysis (PCA) of beta values of all tested CpGs across the participants in the FUSION, Gene SMART, and GSE38291 cohorts.** Males are denoted by green; females are denoted by orange. The FUSION and GSE38291 PCAs were performed on the raw beta values, the Gene SMART PCA was performed beta values adjusted for analysis set (the residuals between the raw beta values and the linear model DNAm~set (2017 vs 2019); given that analysis set dichotomized the raw beta values.


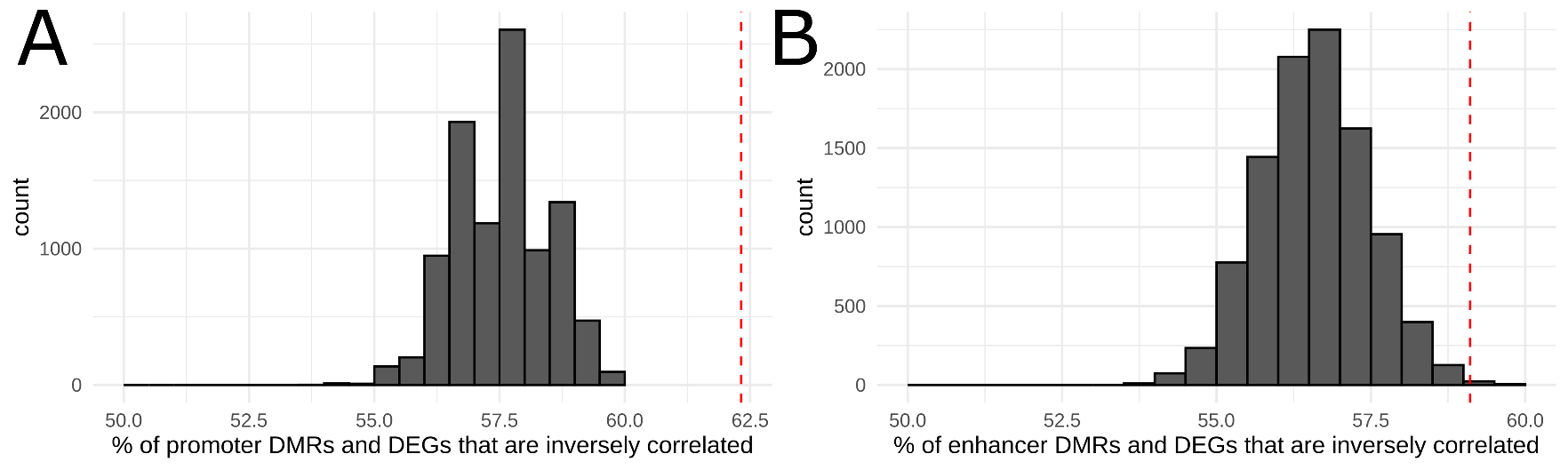


**Supplementary Figure 5. Distribution of the 10,000 random permutations for a negative correlation between DNA methylation and gene expression.** (**A**) Histogram of 10,000 random permutations of DMRs annotated to promoter regions and correlation with GTEx gene expression. Effect sizes of DMRs were randomly shuffled and the resulting correlation with gene expression is plotted. Red dashed line indicated the real percentage of promoter DMRs that are negatively correlated with gene expression. (**B**) Histogram of 10,000 random permutations of DMRs annotated to enhancer regions and correlation with GTEx gene expression. Effect sizes of DMRs were randomly shuffled and the resulting correlation with gene expression is plotted. Red dashed line indicated the real percentage of enhancer DMRs that are negatively correlated with gene expression.


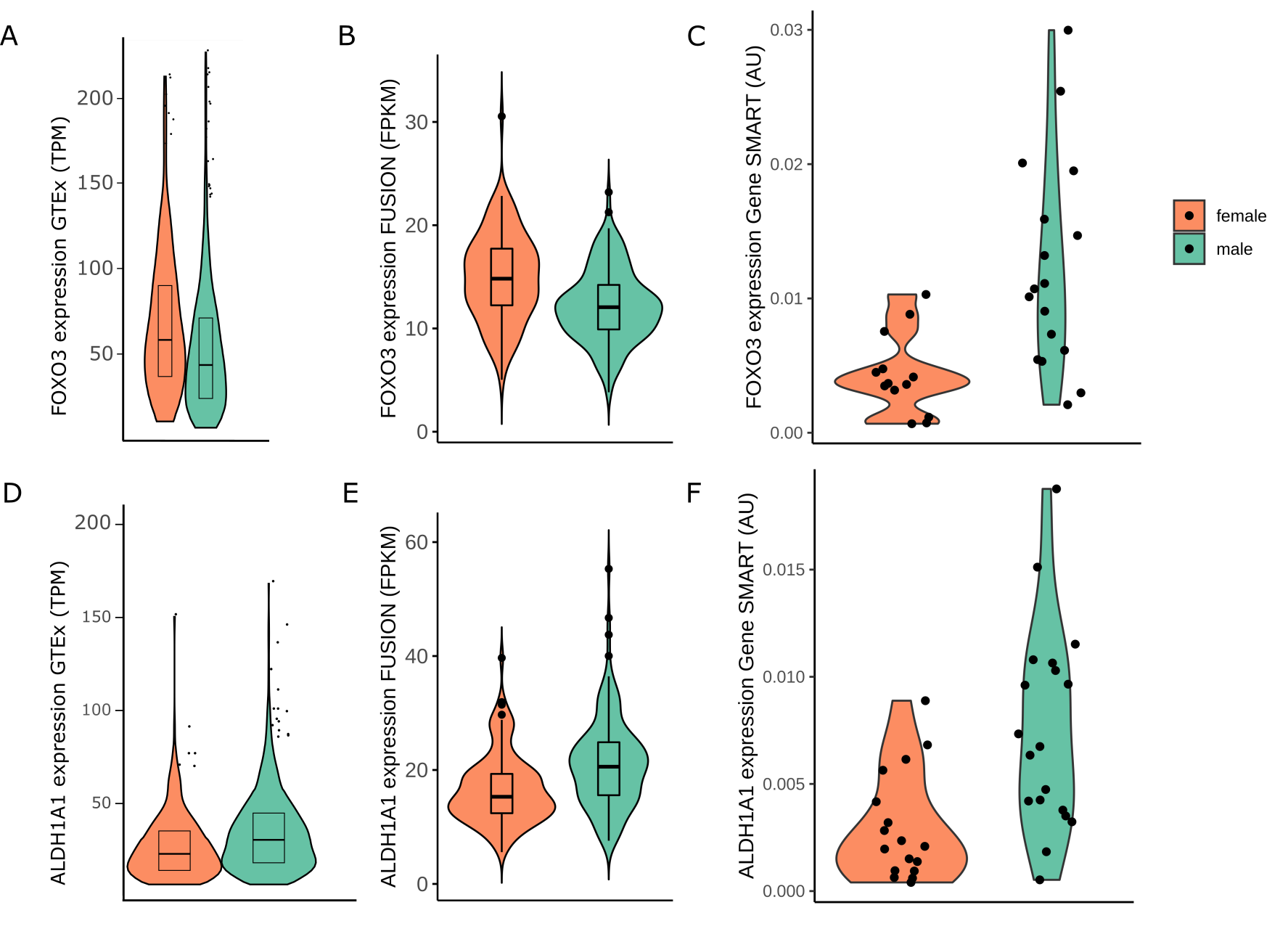


**Supplementary Figure 6. Gene expression for FOXO3 and ALDH1A1 validated in 3 cohorts.** Distribution of FOXO3 expression in males and females from (**A**) the GTEx using RNA-seq (TPM- transcripts per million; n = 803; adapted from GTEx portal) (**B**) the FUSION cohort using RNA-seq (FPKM- fragments per kilobase of transcript per million; n = 274) (**C**) the Gene SMART cohort using qPCR (AU-arbitrary units; 2^-∆Ct^; n = 30). Distribution of ALDH1A1 expression in males and females from (**D**) the GTEx using RNA-seq (TPM; n = 803) (**E**) the FUSION cohort using RNA-seq (FPKM; n = 274) (**F**) the Gene SMART cohort using qPCR (AU; n = 36).


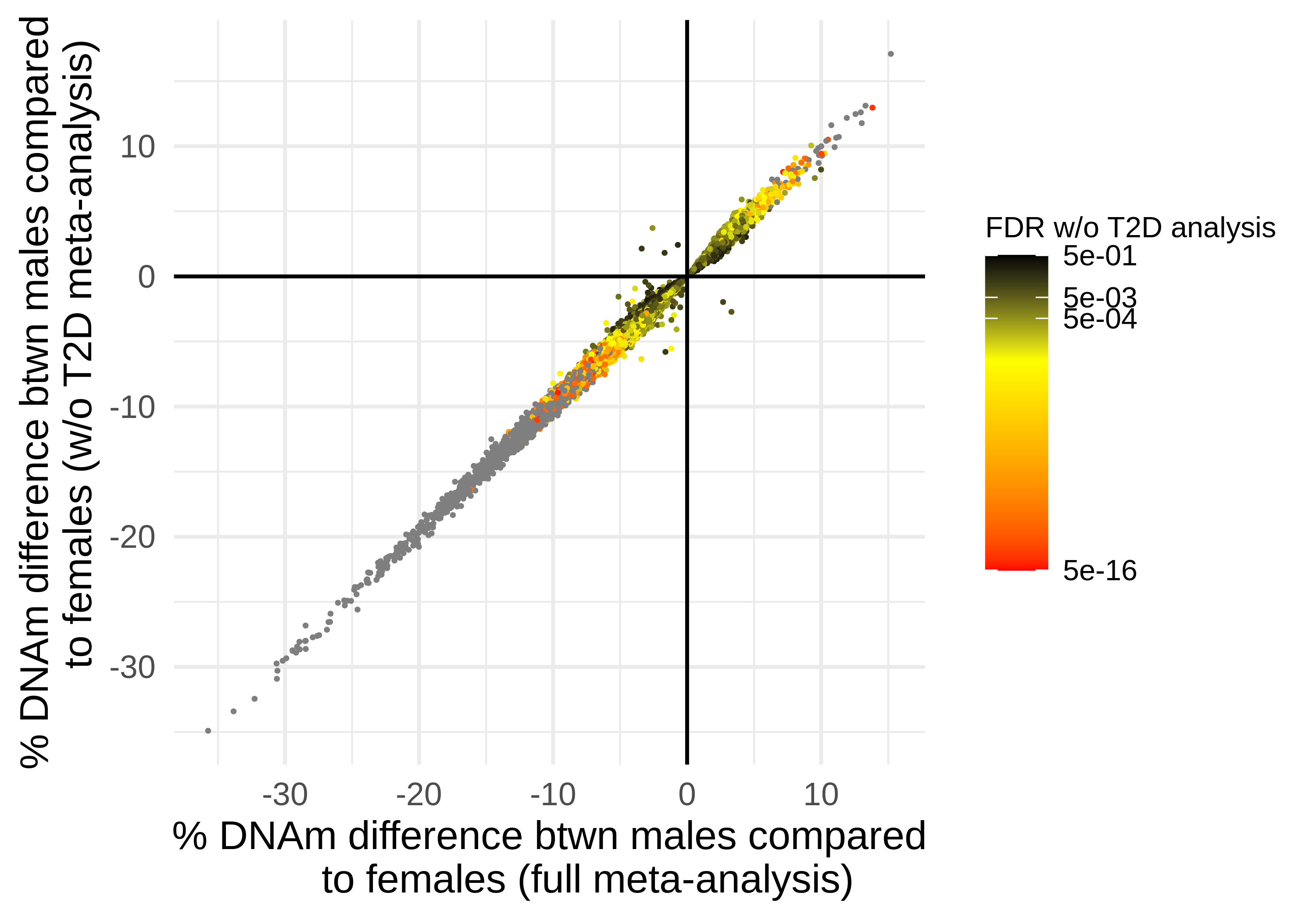


**Supplementary Figure 7. Comparison of results from the full meta-analysis and from a meta-analysis excluding T2D participants in FUSION.** Each point is one of the 56,813 differentially methylated positions (DMPs) discovered in the full meta-analysis (false discovery rate (FDR) < 0.005). To compare results from the full and partial meta-analysis we plotted the effect size (B value percentages) in the full meta-analysis (x-axis) against the effect size of the partial meta-analysis (y-axis). To show whether DMPs remained significant in the partial meta-analysis, we coloured points according to the FDR in the partial meta-analysis.
