# Supplementary figures and images for: Skeletal muscle methylome and transcriptome integration reveals profound sex differences related to muscle function and substrate metabolism
Source: Clin Epigenetics. 2021 Nov 3;13:202. doi: 10.1186/s13148-021-01188-1 (PMC8567658; doi:10.1186/s13148-021-01188-1)

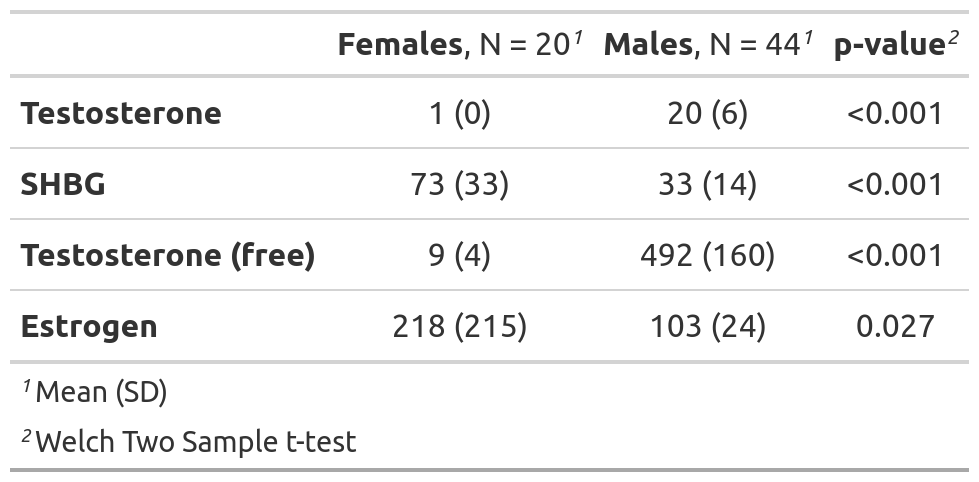

Supplement: Supplementary file 13 — Additional file 13. Concentrations of circulating testosterone (nmol/L), free testosterone (pmol/L), sex hormone-binding globulin (nmol/L), and estrogen (pmol/L) in males and females from the Gene SMART cohort. Includes 20 females (20 at rest before four weeks of exercise training, same 20 at rest after four weeks of exercise training, and six of those at rest before a control period) and 44 males (we did not have data for one male included in the DNA methylation analysis). [file 13148_2021_1188_MOESM13_ESM.png]
